# Onco-lncRNA *HOTAIR* and its functional genetic variants in papillary thyroid carcinoma

**DOI:** 10.1038/srep31969

**Published:** 2016-08-23

**Authors:** Hui Zhu, Zheng Lv, Changming An, Meng Shi, Wenting Pan, Liqing Zhou, Wenjun Yang, Ming Yang

**Affiliations:** 1 Department of Radiation Oncology, Shandong Cancer Hospital affiliated to Shandong University, Shandong Academy of Medical Sciences, Jinan, Shandong Province, China; 2 Cancer Center, The First Affiliated Hospital of Jilin University, Changchun, Jilin Province, China; 3 Department of Head and Neck Surgical Oncology, Cancer Hospital, Chinese Academy of Medical Sciences, Beijing, China; 4 College of Life Science and Technology, Beijing University of Chemical Technology, Beijing, China; 5 Department of Radiation Oncology, Huaian No. 2 Hospital, Huaian, Jiangsu Province, China; 6 Key Laboratory of Fertility Preservation and Maintenance (Ministry of Education), Ningxia Medical University, Yinchuan, Ningxia, China; 7 Shandong Provincial Key Laboratory of Radiation Oncology, Cancer Research Center, Shandong Cancer Hospital affiliated to Shandong University, Shandong Academy of Medical Sciences, Jinan, Shandong Province, China

## Abstract

The role of long noncoding RNA (lncRNA) HOX transcript antisense RNA (HOTAIR) and its functional single nucleotide polymorphisms (SNPs) in papillary thyroid carcinoma (PTC) is still largely unclear. Therefore, we investigated the involvement of lncRNA HOTAIR and its three haplotype-tagging SNPs (htSNPs) in PTC. There was higher expression of *HOTAIR* in PTC tissues compared to normal tissues. A series of gain-loss assays demonstrated that *HOTAIR* acts as a PTC oncogene via promoting tumorigenic properties of PTC cells. Additionally, the functional *HOTAIR* rs920778 genetic variant was a PTC susceptibility SNP. Subjects with the *HOTAIR* rs920778 TT genotype had an odds ratio (OR) of 1.88, 1.25 and 1.61 (*P* = 6.0 × 10^−6^, *P* = 0.028 and *P* = 3.2 × 10^−5^) for developing PTC in Shandong, Jiangsu and Jilin case-control sets compared with subjects with the CC genotype. This statistically significant associations were only found between the rs920778 genetic polymorphism and PTC risk in females but not in males. The allele-specific regulation on *HOTAIR* expression by the rs920778 SNP was confirmed both *in vitro* and *in vivo*. Our results demonstrate that functional SNPs influencing lncRNA regulation may explain a part of PTC genetic basis.

As the most common endocrine malignancy, thyroid carcinoma showed quickly increased morbidity over last two decades. The Chinese Cancer Registry reported that the incidence of thyroid carcinoma is 6.6 per 100,000 individuals in China^1^. Among all patients with thyroid carcinoma, about eighty percents carried papillary thyroid carcinoma (PTC) which was named for its papillary histological architecture. Multiple risk factors of PTC have been identified, such as ionizing radiation, nodular disease of the thyroid and family history^2^. However, not all exposed individuals finally developed PTC, indicating that genetic makeup may also be involved in etiology of PTC^3^. New insights into the pathogenesis of this malignant disease are therefore needed.

Long noncoding RNAs (lncRNAs) comprise a very heterogeneous subclass of RNAs that were firstly described by the genome-wide sequencing of cDNA libraries of the mouse genome^4^. It is becoming clear that a large number of lncRNAs are key regulators of physiology and the processes of diseases such as cancers, despite so often being branded as transcriptional noise^4^. LncRNA HOX transcript antisense RNA (HOTAIR) is coded from the homebox C gene (HOXC) locus and involved in development and progression of multiple malignances^4^,^5^,^6^,^7^,^8^,^9^,^10^,^11^. As one of the trans-regulatory lncRNAs, HOTAIR facilitates aberrant PRC2 function by increasing PRC2 recruitment to the genomic positions of target genes and, thus, promotes malignant transformation and cancer cell metastasis^4^,^5^,^6^,^7^,^8^,^9^,^10^,^11^. In line with this, HOTAIR is upregulated in cancer tissues and overexpression of HOTAIR might be an independent predictor of overall survival and progression-free survival^4^,^5^. All these reports elucidate the involvement of HOTAIR during carcinogenesis. However, it is still largely unclear how HOTAIR is involved in malignant transformation of thyroid cells. Therefore, we examined its expression in tissue specimens of PTC and normal thyroid tissues and elucidated its oncogene nature in PTC cells.

In two previous studies, we reported the functional significance of the lncRNA *HOTAIR* single nucleotide polymorphisms (SNP) in esophageal squamous cell carcinoma (ESCC) and gastric cancer^12^,^13^. We found that the *HOTAIR* rs920778 TT genotype was significantly associated with increased risk of ESCC and gastric cancer^12^,^13^. During inspecting functional relevance of the rs920778 SNP, we identified a novel intronic *HOTAIR* enhancer locating between +1719 bp and +2353 bp from the transcriptional start site through dual luciferase reporter gene assays. Moreover, there is an allelic regulation of rs920778 on lncRNA HOTAIR expression via this enhancer in both ESCC and gastric cancer, with higher HOTAIR expression among T allele carriers. To date, little or nothing has been known on how *HOTAIR* SNPs are involved in etiology of PTC. On the basis of our previous findings, we hypothesized that the functional genetic variants in the *HOTAIR* gene may affect HOTAIR expression and influence consequential risk of developing PTC. To test this hypothesis, we genotyped three *HOTAIR* haplotype-tagging SNPs (htSNP) in three large independent case-control sets to examine the association between *HOTAIR* genotypes and PTC risk. The regulatory functions of the PTC susceptibility SNP rs920778 on HOTAIR expression was then investigated in PTC cell lines and in patient tissue specimens.

## Materials and Methods

### Real-time analyses of lncRNA HOTAIR

Total cellular RNA was isolated from culture cells, sixty pairs of PTC tissues and normal specimens adjacent to the tumors with TRIzol Reagent (Invitrogen, US) and treated with RNase-Free DNase to remove genomic DNA (Invitrogen). Each RNA sample was reverse transcribed into cDNA with Revert Ace kit (TOYOBO, Osaka, Japan). Relative gene expression of lncRNA HOTAIR in tissues was analyzed using the SYBR-Green real-time quantitative PCR method. The expression of HOTAIR of individual specimen was calculated relative to expression of *β-actin* mRNA using the 2^−dCt^ method as described previously^12^,^13^,^14^,^15^. All qPCR was carried out using the ABI 7500 real-time PCR system in triplicates.

### Cell proliferation assays

Human PTC BCPAP or HEK293 cells were cultured in RPMI 1640 medium (Invitrogen) supplemented with penicillin-streptomycin and 10% fetal bovin serum (Hyclone, US) at 37 °C with 5% CO_2_. Cells were seeded in a 12-well plate at a density of 10^5^ cells per well. BCPAP or HEK293 cells were transfected with the *HOTAIR* expression construct or pcDNA3.1 vector with Lipofectamine 2000 (Invitrogen). In the gene silencing assays, different concentrations of negative control RNA (NC) or HOTAIR siRNAs (siH-1 and siH-2) (Genepharma, China) were transfected into BCPAP cells combined with Lipofectamine RNAi Max (Invitrogen). Cells were then harvested by trypsin digestion, washed by cold PBS twice, dyed with trypan blue and counted under microscopy at 24 h, 48 h and 72 h after transfection.

### Colony formation assays

A total of 5000 BCPAP or HEK293 cells per well were seeded into a 6-well cell culture plate and transfected with the *HOTAIR* expression construct or pcDNA3.1 vector. After 13 days, cells were washed with cold PBS twice and fixed with 3.7% formaldehyde.

### Study case-control sets

This study consisted of three case-control sets: (a) Shandong set: 600 patients with PTC Shandong Cancer Hospital (Jinan, Shandong Province, China) and sex- and age-matched (±5 years) 600 controls. (ii) Jiangsu set: 1000 cases with PTC from Huaian No. 2 Hospital (Huaian, Jiangsu Province, China) and sex- and age-matched (±5 years) 1000 healthy controls. (iii) Jilin set: 800 PTC patients from The First Affiliated Hospital of Jilin University (Changchun, Jilin Province, China) and 800 sex- and age-matched healthy controls. Sixty pairs of PTC specimens and thyroid normal tissues adjacent to the tumors were obtained from surgically removed specimens of patients in Shandong Cancer Hospital and Huaian No. 2 Hospital. All individuals were ethnic Han Chinese. The detailed information on characteristics of PTC cases and controls can be found in Supplementary Table 1. This study was approved by the institutional Review Boards of Shandong Cancer Hospital, Huaian No. 2 Hospital and The First Affiliated Hospital of Jilin University. At recruitment, the written informed consent was obtained from each subject. The methods were carried out in accordance with the relevant guidelines.

### *HOTAIR* SNP genotyping

Three *HOTAIR* htSNPs (rs920778, rs1899663, and rs4759314) were selected and genotyped as described previously^12^,^13^. SNP genotyping was performed without knowledge of patient or control status. A 15% random sample was reciprocally tested by different individuals, and the reproducibility was 99.7%.

### Dual luciferase reporter assay

The intron 2 region of *HOTAIR* (from +1463 bp to +2353 bp, relative to the transcription start site) was amplified with human genomic DNA from healthy controls to build intronic enhancer reporter constructs as described previously^12^,^13^. The p-C construct (the rs920778C allele) and p-T (the rs920778T allele) constructs were identical, except for the different allele at the rs920778 polymorphic site. The reporter gene constructs pGL3-Basic, p-C, or p-T as well as pRL-SV40 (Luciferase Assay System; Promega) were co-transfected into PTC cell line BCPAP cells or HEK293 cells. Both firefly luciferase activity and renilla luciferase activity were measured at 48 h after transfection using a dual luciferase assay system (Promega) as previously described^12^,^13^,^14^,^15^. For each plasmid construct, three independent transfection experiments were performed, and each was done in triplicates.

### Statistics

Pearson’s χ^2^ test was used to examine the differences in demographic variables and genotype distributions of *HOTAIR* htSNPs between PTC cases and controls. Associations between *HOTAIR* genotypes and PTC risk were estimated by odds ratio (OR) and their 95% confidence intervals (CIs) computed using the unconditional logistic regression model. All ORs were adjusted for age and sex, where it was appropriate. A *P* value of less than 0.05 was used as the criterion of statistical significance. All statistical tests were two-sided and performed with the SPSS software package (Version 16.0, SPSS Inc., Chicago, IL).

## Results

**Figure 1:**
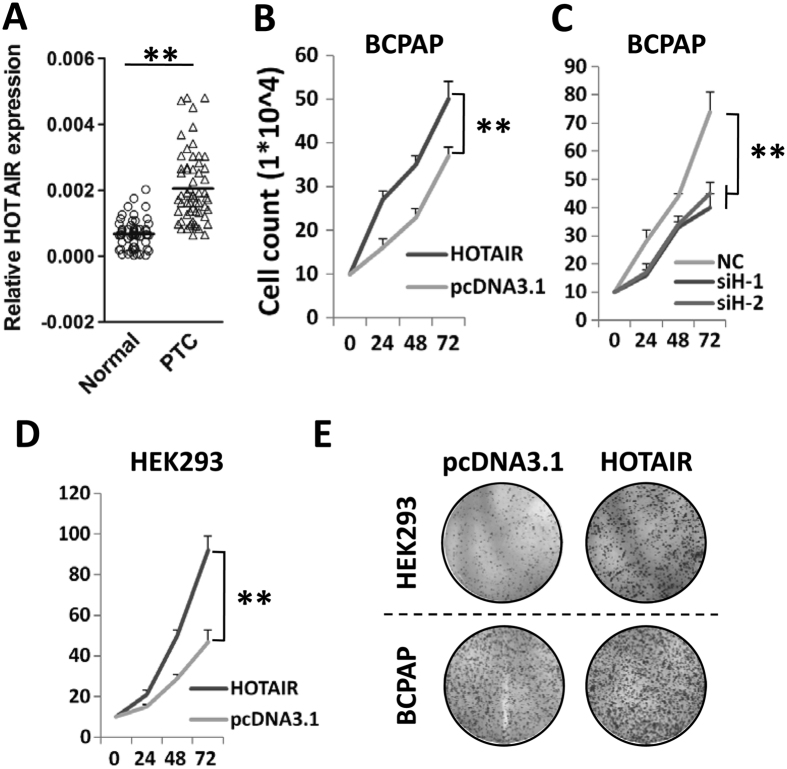
*HOTAIR* acts as an oncogene in PTC. (**A**) *HOTAIR* RNA expression detected in 60 pairs of PTC tissue specimens and normal tissues. (**B**) Ectopically expressed *HOTAIR* could significantly stimulate cell viability in BCPAP cells. (**C**) Depletion of *HOTAIR* expression in BCPAP cells significantly inhibited cell proliferation. (**D**) Over-expression of lncRNA *HOTAIR* could significantly promote cell growth of HEK293 cells. (**E**) Colony formation assays. At the 13rd day after transfection of the *HOTAIR* expression construct or pcDNA3.1 vector, cells were washed with cold PBS twice and fixed with 3.7% formaldehyde. All experiments were performed in triplicates in three independent transfection experiments and each value represents mean ± SD. **P < 0.01.

By detecting *HOTAIR* RNA expression in sixty paired PTC and normal tissues, we found higher expression of HOTAIR in PTC tissues compared to normal tissues (*P* < 0.05) (Fig. 1A). In BCPAP or HEK293 cells, ectopic expression of lncRNA HOTAIR significantly promoted cell proliferation (Fig. 1B,C). On the contrary, depletion of endogenous *HOTAIR* expression significantly suppresses PTC cell proliferation (Fig. 1D). In line with cell viability assays, HOTAIR is able to promote colony formation in both BCPAP and HEK293 cells (Fig. 1E). These results are consistent to the role of HOTAIR in other malignances, indicating the oncogene nature of *HOTAIR* in PTC development.

Considering the important involvement of genetic polymorphisms in regulating expression of *HOTAIR*, we next investigated the association between three *HOTAIR* htSNPs (rs920778 C>T, rs1899663 G>T, and rs4759314 A>G) and PTC susceptibility in a case-control design. A total of three case-control sets were included in the current study. There are no statistically significant differences between PTC cases and controls for all three sets in terms of median age and sex distributions (all *P* > 0.05), indicating that the frequency matching was adequate (Supplementary Table 1). Allele frequencies and genotype distributions of *HOTAIR* htSNPs in cases and controls from the Shandong training set are showed in Table 1. The allele frequencies for rs920778 T, rs1899663 T and rs4759314 G were 0.304, 0.138, and 0.052 in cases and 0.206, 0.166, and 0.041 in controls in Shandong training case-control set. All observed genotype frequencies in both cases and controls conform to Hardy-Weinberg equilibrium. Distributions of the rs920778, rs1899663 and rs4759314 genotypes were compared between cases and controls. Frequencies of rs920778 CC, CT, and TT genotypes among PTC cases differed significantly from those among controls (χ^2^ = 30.68, *P* = 3.0 × 10^−8^, *df* = 2), with the frequency of TT homozygote being significantly higher among patients than among controls (8.8% vs. 3.2%). However, no statistically significant differences of rs1899663 and rs4759314 genotypes were observed between cases and controls (both *P* > 0.05). As a result, we did not do further investigations on these two genetic polymorphisms.

Associations between genotypes of *HOTAIR* rs920778 and PTC risk were firstly estimated in Shandong training set (Table 2). The rs920778 T allele was shown to be risk allele. Subjects with the rs920778 TT genotype had an OR of 1.88 (95% CI = 1.43–2.47, *P* = 6.0 × 10^−6^) for developing PTC in Shandong population compared with subjects with the CC genotype. This association was successfully validated in the other two independent case-control sets (Jiangsu set: OR = 1.25, 95% CI = 1.02–1.53, *P* = 0.028; Jilin set: OR = 1.61, 95% CI = 1.28–2.01, *P* = 3.2 × 10^−5^) (Table 2). Interestingly, the rs920778 CT genotype was also significantly associated with increased PTC risk compared to the rs920778 CC genotype in all three sets (Shandong: OR = 1.60, 95% CI = 1.26–2.03, *P* = 1.2 × 10^−4^; Jiangsu: OR = 1.22, 95% CI = 1.01–1.47, *P* = 0.038; Jilin: OR = 1.30, 95% CI = 1.06–1.60, *P* = 0.014). In the pooled analyses, the rs920778 CT or TT genotype carriers showed a 1.40-fold or 1.66-fold increased PTC risk compared with the CC genotype carriers (95% CI = 1.21–1.63, *P* = 7.8 × 10^−6^ or, 95% CI = 1.41–1.95, *P* = 8.1 × 10^−10^) (Table 2). The PTC risk associated with the rs920778 genetic variant was further examined by stratifying for age and sex using the combined data of three case-control sets (Table 3). Statistically significant associations were found between the rs920778 genetic polymorphism and PTC risk only in females but not in males, with OR of CT genotype or TT genotype equaling to 1.46 (95% CI = 1.22–1.73, *P* = 2.1 × 10^−5^) or 1.75 (95% CI = 1.45–2.10, *P* = 2.4 × 10^−9^). Significant associations between rs920778 CT or TT genotype and PTC risk were observed in all age-stratified groups (all *P* < 0.05) (Table 3).

**Figure 2:**
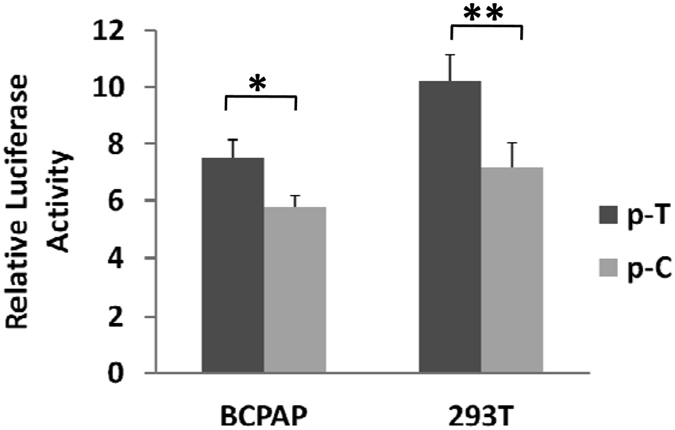
Transient luciferase reporter gene assays with constructs containing different rs920778 allele of *HOTAIR* in BCPAP cells (**A**) or 293T cells (**B**). pRL-SV40 were cotransfected with these contructs to standardize transfection efficiency. Fold-changes were detected by defining the luciferase activity of cells co-transfected with pGL3-basic as 1. All experiments were performed in triplicates in three independent transfection experiments and each value represents mean ± SD. Compared with pGL3-Basic transfected cells, *P < 0.05; **P < 0.01.

Our previous studies demonstrated that the PTC susceptibility SNP rs920778 is a functional genetic variant located within a potential *HOTAIR* enhancer^12^,^13^. Therefore, we investigated its regulatory potential in PTC using reporter gene assays (Fig. 2). Both BCPAP cells or 293T cells transfected with *HOTAIR* rs920778T allelic reporter construct (p-T) showed significantly elevated luciferase activities compared to cells expressing rs920778C allelic reporter construct (p-C) (both *P* < 0.05) (Fig. 2).

**Figure 3:**
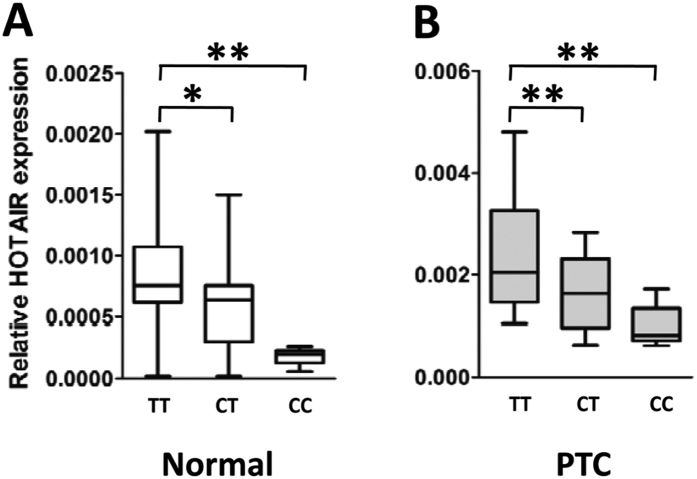
*HOTAIR* RNA expression in normal thyroid tissue specimens and PTC tissues grouped by rs920778 genotypes. The expression of individual *HOTAIR* RNA was calculated relative to expression of *β-actin* mRNA using the 2^−dCt^ method. *P < 0.05; **P < 0.01.

We next examined whether the PTC susceptibility SNP rs920778 has an allele-specific effect on *HOTAIR* expression using PTC tissues. As shown in Fig. 3, we found that subjects with the rs920778 CT or TT genotype had significantly higher *HOTAIR* RNA levels (mean ± SD) than those with the CC genotypes in normal tissues (CC: 0.0002 ± 0.0001 [*n* = 31], CT: 0.0006 ± 0.0003 [*n* = 24], TT: 0.0008 ± 0.0004 [*n* = 5]; CC vs. CT: *P* = 0.045, CC vs. TT: *P* = 0.045). Similar results were observed when the *HOTAIR* RNA levels were compared between rs920778 CT or TT and CC genotypes in PTC tissues (CC: 0.0009 ± 0.0004 [*n* = 31], CT: 0.0016 ± 0.0006 [*n* = 24], TT: 0.0025 ± 0.0012 [*n* = 5]; CC vs. CT: *P* = 0.005, CC vs. TT: *P* = 0.009).

## Discussion

In the current study, we investigated the oncogene role of lncRNA *HOTAIR* in PTC and the association between *HOTAIR* htSNPs and PTC risk through a two stage case-control approach. To the best of our knowledge, this is the first study to examine how lncRNA *HOTAIR* and its genetic polymorphisms play their role in PTC development. We found that the functional *HOTAIR* rs920778 SNP was a PTC susceptibility polymorphism in Chinese. Dual luciferase reporter gene assays and tissue data indicated that the rs920778 SNP in the gene enhancer impacts lncRNA HOTAIR expression in an allele-specific way, which is consistent to our previous findings in gastric cancer and esophageal cancer^12^,^13^.

We and others have identified multiple lncRNAs involved in PTC tumorigenesis and progression. Yoon H *et al*. identified a novel noncoding RNA gene, NAMA (noncoding RNA associated with MAP kinase pathway and growth arrest), that is downregulated in PTC with BRAF mutation and associated with growth arrest^16^. Jendrzejewski J *et al*. described a unique lncRNA named Papillary Thyroid Carcinoma Susceptibility Candidate 3 (PTCSC3) located 3.2 kb downstream of rs944289 at 14q.13.3 which is a GWAS identified PTC susceptibility region^17^. LncRNA PTCSC3 is involved in PTC development by modulating S100A4 gene expression^18^. Interestingly, a novel lncRNA papillary thyroid cancer susceptibility candidate 2 (PTCSC2) gene was found locating in another 9q22 PTC risk loci^19^. We performed a whole exome sequencing in 91 pairs PTC samples and identified a lncRNA GAS8-AS1 as novel PTC driver alternations^20^. In addition, several other lncRNAs including FAL1, LOC100507661, H19, ENST00000537266 and ENST00000426615 are involved in PTC development^21^,^22^,^23^,^24^. However, it is still largely unknown if onco-lncRNA HOTAIR also plays a part in PTC. We found that elevated HOTAIR can promote PTC cell proliferation and silencing its expression inhibits cell growth, suggesting its oncogene nature in PTC. Our results demonstrated that *HOTAIR* rs920778T allele is a PTC risk allele and is associated with significantly increased HOTAIR RNA expression. Considering the oncogene role of *HOTAIR* in PTC, the association might be biological rational.

Multiple limitations exist in our hospital-based case-control study. For example, the hospital-based design might lead to inherent selection bias of PTC cases. Therefore, the results of our current study warrant to be validated in a population-based prospective study in the future. Moreover, the potential clinical translation of this SNP might be compromised due to the relatively low allele frequency of the rs920778 TT genotype.

In summary, we identified the involvement of lncRNA *HOTAIR* in PTC development and a functional PTC susceptibility SNP rs920778 in Chinese Han populations. The rs920778 C>T polymorphism leads to increased HOTAIR RNA expression, which might be the underlying mechanism in conferring PTC susceptibility. These data support the hypothesis that functional SNPs influencing lncRNA regulation may explain a part of PTC genetic basis^17^,^18^,^19^.

## Additional Information

**How to cite this article**: Zhu, H. *et al*. Onco-lncRNA *HOTAIR* and its functional genetic variants in papillary thyroid carcinoma. *Sci. Rep.* **6**, 31969; doi: 10.1038/srep31969 (2016).

## Supplementary Material

Supplementary Information

## Figures and Tables

**Table 1:** Associations between candidate SNPs in *HOTAIR* and PTC risk in Shandong case-control set (Training set).

#	Identity	Location	Position^1^	Case	Common genotype %	Heterozygous genotype %	Rare genotype %	OR^2^ (95% CI) for heterozygote	*P*	OR^2^ (95% CI) for rare genotype	*P*
1	rs920778	Intron 2	52646499	PTC	288(48.0)	259(43.2)	53(8.8)	1.60(1.26–2.03)	1.2 × 10^−4^	1.88(1.43–2.47)	6.0 × 10^−6^
	(C>T)			Control	372(62.0)	209(34.8)	19(3.2)				
2	rs1899663	Intron 2	52647261	PTC	442(73.6)	151(25.2)	7(1.2)	0.81(0.63–1.04)	0.103	0.72(0.45–1.15)	0.716
	(G>T)			Control	413(68.8)	175(29.2)	12(2.0)				
3	rs4759314	Intron 1	52648102	PTC	540(90.0)	58(9.7)	2(0.3)	1.34(0.99–2.01)	0.163	1.01(0.38–2.70)	0.986
	(A>G)			Control	553(92.2)	45(7.5)	2(0.3)				

Note: PTC, papillary thyroid carcinoma; OR, odds ratio; CI, confidence interval.

^1^Position in NCBI build 36.

^2^Data were calculated by unconditional logistic regression, adjusted of age and sex.

**Table 2:** Genotype frequencies of the *HOTAIR* rs920778 C>T polymorphism among PTC cases and controls and their association with PTC risk.

Studies	Genotypes	Cases No. (%)	Controls No. (%)	OR^1^ (95% CI)	*P*^1^
		*n = 600*	*n = 600*		
Shandong set	CC	288(48.0)	372(62.0)	1.00 (Reference)	
	CT	259(43.2)	209(34.8)	1.60(1.26–2.03)	1.2 × 10^−4^
	TT	53(8.8)	19(3.2)	1.88(1.43–2.47)	6.0 × 10^−6^
		*n* = 1000	*n* = 1000		
Jiangsu set	CC	553(55.3)	608(60.8)	1.00 (Reference)	
	CT	385(38.5)	348(34.8)	1.22(1.01–1.47)	0.038
	TT	62(6.2)	44(4.4)	1.25(1.02–1.53)	0.028
		*n* = 800	*n* = 800		
Jilin set	CC	416(52.0)	485(60.6)	1.00 (Reference)	
	CT	316(39.5)	284(35.5)	1.30(1.06–1.60)	0.014
	TT	68(8.5)	31(3.9)	1.61(1.28–2.01)	3.2 × 10^−5^
		*n* = 2400	*n* = 2400		
Pooled	CC	1257(52.4)	1465(61.0)	1.00 (Reference)	
	CT	960(40.0)	841(35.0)	1.40(1.21–1.63)	7.8 × 10^−6^
	TT	183(7.6)	94(4.0)	1.66(1.41–1.95)	8.1 × 10^−^1^0^

Note: PTC, papillary thyroid carcinoma; OR, odds ratio; CI, confidence interval.

^1^Data were calculated by logistic regression with adjustment for age and sex.

**Table 3:** Risk of PTC associated with *HOTAIR* rs920778 C>T genotypes by age and sex.

Variable	rs920778 C>T						
	CC^1^	CT^1^	OR^2^ (95% CI)	*P*	TT^1^	OR^2^ (95% CI)	*P*
Sex							
Male	300/388	271/230	1.26(0.95–1.69)	0.114	35/25	1.37(0.97–1.94)	0.074
Female	957/1077	689/611	1.46(1.22–1.73)	2.1 × 10^−5^	148/69	1.75(1.45–2.10)	2.4 × 10^−9^
Age (year)							
≤47	620/722	471/448	1.24(1.01–1.53)	0.045	99/45	1.59(1.29–1.96)	1.6 × 10^−5^
>47	637/743	489/393	1.88(1.49–2.37)	1.0 × 10^−7^	84/49	1.46(1.16–1.83)	0.001

Note: PTC, papillary thyroid carcinoma; OR, odds ratio; CI, confidence interval.

^1^Number of case patients with genotype/number of control subjects with genotype.

^2^Data were calculated by logistic regression, adjusted for sex and age, where it was appropriate.
