# Supplementary material for: Onco-lncRNA HOTAIR and its functional genetic variants in papillary thyroid carcinoma
Source: Sci Rep. 2016 Aug 23;6:31969. doi: 10.1038/srep31969 (PMC4994070; doi:10.1038/srep31969)
Supplement: Supplementary Information [file srep31969-s1.doc]

**Onco-lncRNA *HOTAIR* and its functional genetic variants in papillary thyroid carcinoma**

Hui Zhu^1,†^; Zheng Lv^2^,**^†^**; Changming An^3^; Meng Shi^4^; Wenting Pan^4^; Liqing Zhou^5^; Wenjun Yang^6^; Ming Yang^7,*^

**Authors’ affiliations:** ^1^Department of Radiation Oncology, Shandong Cancer Hospital affiliated to Shandong University, Shandong Academy of Medical Sciences, Jinan, Shandong Province, China; ^2^Cancer Center, The First Affiliated Hospital of Jilin University, Changchun, Jilin Province, China; ^3^Department of Head and Neck Surgical Oncology, Cancer Hospital, Chinese Academy of Medical Sciences, Beijing, China; ^4^College of Life Science and Technology, Beijing University of Chemical Technology, Beijing, China; ^5^Department of Radiation Oncology, Huaian No. 2 Hospital, Huaian, Jiangsu Province, China; ^6^Key Laboratory of Fertility Preservation and Maintenance (Ministry of Education), Ningxia Medical University, Yinchuan, Ningxia, China; ^7^Shandong Key Laboratory of Radiation Oncology, Cancer Research Center, Shandong Cancer Hospital affiliated to Shandong University, Shandong Academy of Medical Sciences, Jinan, Shandong Province, China.

^*^**Correspondence to:** Ming Yang, PhD, Professor, Shandong Key Laboratory of Radiation Oncology, Cancer Research Center, Shandong Cancer Hospital affiliated to Shandong University，Shandong Academy of Medical Sciences, Jinan 250117, Shandong Province, China. Tel & Fax: 86531-67626536; E-mail: aaryoung@yeah.net.

^†^**Note:** Hui Zhu and Zheng Lv contribute equally to this work.

**Supplementary Table 1.** Distribution of selected characteristics among PTC cases and controls

| Variable | Shandong case-control set  (Discovery set) | | |  | Jiangsu case-control set  (Validation set 1) | | |  | Jilin case-control set  (Validation set 2) | | |  |
| --- | --- | --- | --- | --- | --- | --- | --- | --- | --- | --- | --- | --- |
|  | Cases | Controls | *P*-value ^a^ |  | Cases | Controls | *P*-value^a^ |  | Cases | Controls | *P*-value^a^ |  |
|  | No. (%) | No. (%) |  |  | No. (%) | No. (%) |  |  | No. (%) | No. (%) |  |  |
|  | 600 | 600 |  |  | 1000 | 1000 |  |  | 800 | 800 |  |  |
| Sex |  |  | 0.601 |  |  |  | 0.223 |  |  |  | 0.774 |  |
| Male | 155(25.8) | 163(27.2) |  |  | 251(25.1) | 275(27.5) |  |  | 200(25.0) | 205(25.6) |  |  |
| Female | 445(74.2) | 437(72.8) |  |  | 749(74.9) | 725(72.5) |  |  | 600(75.0) | 595(74.4) |  |  |
| Age (year)^2^ |  |  | 0.149 |  |  |  | 0.823 |  |  |  | 0.453 |  |
| ≤45(or 48) | 278(46.3) | 303(50.5) |  |  | 526(52.6) | 521(52.1) |  |  | 411(51.4) | 426(53.2) |  |  |
| >45(or 48) | 322(53.7) | 297(49.5) |  |  | 474(47.4) | 479(47.9) |  |  | 389(48.6) | 374(46.8) |  |  |
|  |  |  |  |  |  |  |  |  |  |  |  |  |

Note: PTC, papillary thyroid carcinoma.

^1^Two-sided χ^2^ test.

^2^Median ages of cases for Shandong case-control set, Jiangsu case-control set and Jilin case-control set are 45, 48 and 48 years.
